# Endothelial ACKR3 drives atherosclerosis by promoting immune cell adhesion to vascular endothelium

**DOI:** 10.1007/s00395-022-00937-4

**Published:** 2022-06-08

**Authors:** Selin Gencer, Yvonne Döring, Yvonne Jansen, Soyolmaa Bayasgalan, Yi Yan, Mariaelvy Bianchini, Ismail Cimen, Madeleine Müller, Linsey J. F. Peters, Remco T. A. Megens, Philipp von Hundelshausen, Johan Duchene, Patricia Lemnitzer, Oliver Soehnlein, Christian Weber, Emiel P. C. van der Vorst

**Affiliations:** 1 Institute for Cardiovascular Prevention (IPEK), Ludwig-Maximilians University Munich, Pettenkoferstr 9, 80336 Munich, Germany; 2 DZHK (German Center for Cardiovascular Research), Partner Site Munich Heart Alliance, Munich, Germany; 3 Department of Angiology, Swiss Cardiovascular Center, Inselspital, Bern University Hospital, University of Bern, Bern, Switzerland; 4 Interdisciplinary Center for Clinical Research (IZKF), Institute for Molecular Cardiovascular Research (IMCAR), RWTH Aachen University, Aachen, Germany; 5 Department of Pathology, Cardiovascular Research Institute Maastricht (CARIM), Maastricht University, Maastricht, The Netherlands; 6 Department of Biochemistry, Cardiovascular Research Institute Maastricht (CARIM), Maastricht University Medical Centre, Maastricht, The Netherlands; 7 Institute for Experimental Pathology (ExPat), Center for Molecular Biology of Inflammation, WWU Münster, Münster, Germany; 8 Department of Physiology and Pharmacology (FyFa), Karolinska Institutet, Stockholm, Sweden; 9 Munich Cluster for Systems Neurology (SyNergy), Munich, Germany

**Keywords:** Atherosclerosis, Endothelium, Inflammation, Vascular biology, ACKR3

## Abstract

Atherosclerosis is the foundation of potentially fatal cardiovascular diseases and it is characterized by plaque formation in large arteries. Current treatments aimed at reducing atherosclerotic risk factors still allow room for a large residual risk; therefore, novel therapeutic candidates targeting inflammation are needed. The endothelium is the starting point of vascular inflammation underlying atherosclerosis and we could previously demonstrate that the chemokine axis CXCL12–CXCR4 plays an important role in disease development. However, the role of ACKR3, the alternative and higher affinity receptor for CXCL12 remained to be elucidated. We studied the role of arterial ACKR3 in atherosclerosis using western diet-fed *Apoe*^*−/−*^ mice lacking *Ackr3* in arterial endothelial as well as smooth muscle cells. We show for the first time that arterial endothelial deficiency of ACKR3 attenuates atherosclerosis as a result of diminished arterial adhesion as well as invasion of immune cells. ACKR3 silencing in inflamed human coronary artery endothelial cells decreased adhesion molecule expression, establishing an initial human validation of ACKR3’s role in endothelial adhesion. Concomitantly, ACKR3 silencing downregulated key mediators in the MAPK pathway, such as ERK1/2, as well as the phosphorylation of the NF-kB p65 subunit. Endothelial cells in atherosclerotic lesions also revealed decreased phospho-NF-kB p65 expression in ACKR3-deficient mice. Lack of smooth muscle cell-specific as well as hematopoietic ACKR3 did not impact atherosclerosis in mice. Collectively, our findings indicate that arterial endothelial ACKR3 fuels atherosclerosis by mediating endothelium-immune cell adhesion, most likely through inflammatory MAPK and NF-kB pathways.

**Supplementary Information:**

The online version contains supplementary material available at 10.1007/s00395-022-00937-4.

## Introduction

Atherosclerosis is a serious disease silently progressing in large arteries and it can manifest in potentially fatal cardiovascular complications [17]. Risk factors, such as high blood pressure and surplus of circulating lipid levels damage the vascular lining made up of endothelial cells and ignite an inflammatory process [2, 62]. Targeting the immune response involved in the development of atherosclerosis may prove to be a robust treatment option, as the inflammatory processes fuel the expansion of lesions. This is also evident from the results of the CANTOS trial, revealing that anti-inflammatory treatment targeting IL-1ß mediated immune responses reduced cardiovascular risk independent of lipid levels [46]. Atherosclerotic inflammatory processes primarily involve the vascular wall; for example, hypertension leads to circumferential stress on vascular endothelium and hyperlipidemia leads to the invasion of lipids through the vascular endothelium, both of which damage the vessel wall and elicit endothelial cell inflammation [3, 23]. In this process, endothelial cells release cytokines and chemokines, such as CXCL12, to recruit immune cells to the site of injury [19]. Subsequently, immune cells infiltrate the vessel wall to reach the sub-endothelial space and combat the source of inflammation, such as oxidizing lipids [24]. Endothelial cells majorly contribute to this process through the expression of adhesion molecules allowing immune cells to adhere to the vascular wall and eventually transmigrate into the sub-endothelial space [39]. All in all, the endothelium plays a critical role in the onset as well as the development of atherosclerosis and is, therefore, an interesting target for atherosclerosis therapy.

Previously, we established that endothelial cell-derived inflammatory chemokine CXCL12 drives atherosclerosis as its deficiency decreased lesion sizes in the aortas of hyperlipidemic mice [14]. CXCL12 is best known for its roles in the regulation of homing and mobilization of hematopoietic progenitor and stem cells, as well as leukocytes [13]. Moreover, this chemokine is upregulated during inflammation and hyperlipidemia, which are major contributors to cardiovascular risk. CXCL12 was also established as a significant contributor to cardiovascular diseases (CVDs) by genome-wide association studies (GWAS) revealing a strong association of its genetic locus and CVDs [16]. This finding marks the two receptors of CXCL12, CXCR4 and ACKR3 (previously known as CXCR7) as promising investigation targets. Both receptors are known as fundamental members of a healthy cardiac system, as mice lacking these receptors die prenatally as a result of lethal cardiac deformities [22, 53]. Our research group could already reveal that arterial CXCR4 is protective against atherosclerosis by maintaining the integrity of the arterial wall [12]. However, the role of arterial ACKR3 in atherosclerosis is still unclear and therefore remains as a big gap in cardiovascular research. ACKR3 is an atypical chemokine receptor, which lacks G-protein signaling due to structural differences compared to conventional chemokine receptors (G-protein-coupled receptors) [20]. Moreover, ACKR3 has a tenfold higher affinity to CXCL12 compared to CXCR4 and may, therefore, elicit stronger effects in response to CXCL12. Although ACKR3 was reported to be a simple decoy receptor for CXCL12 in the early years of its research [40], recent studies proved its ability to induce cell signaling and even cell chemotaxis [1, 31, 33].

As vascular CXCL12 and CXCR4 were shown to significantly contribute to atherosclerosis, the aim of this study was to decipher the role of arterial ACKR3 in atherosclerosis, which is an important missing link in this chemokine-axis. We further evaluate the role of ACKR3 in the hematopoietic compartment due to the importance of the contribution of immune cells to atherosclerosis as well as the important regulatory roles of CXCL12 in the hematopoietic cell compartment, as mentioned above. To this end, we generated mice that lack *Ackr3* specifically in arterial endothelial cells, smooth muscle cells (SMC) or in the hematopoietic compartment on an *Apoe*^*−/−*^ background and investigated atherosclerosis development by western diet (WD) feeding.

## Methods

### Mice

*Ackr3-floxed (Ackr3*^*fl/fl*^*)* mice provided by ChemoCentryx, Inc. (Mountain View, CA) were crossed with Apolipoprotein E deficient (*Apoe*^*−/−*^) mice to generate *Ackr3*^*fl/fl*^*Apoe*^*−/−*^ mice (> 10 generations). For tamoxifen-inducible, arterial endothelial cell-specific deletion of *Ackr3*, *Ackr3*^*fl/fl*^*Apoe*^*−/−*^ mice were crossed with BmxCreER^T2^ expressing mice (*BmxCre*) kindly provided by Dr. R. Adams (MPI Münster, Germany). Smooth muscle cell (SMC) specific deletion of *Ackr3* was achieved by crossing *Ackr3*^*fl/fl*^*Apoe*^*−/−*^ mice with SmmhcCreER^T2^ expressing mice (*SmmhcCre*) kindly provided by Dr. S. Offermanns (MPI Bad Nauheim, Germany). The knockouts were induced with daily tamoxifen injections (Sigma; 1.5 mg per 20 g body weight, dissolved in corn oil) for 5 consecutive days, followed by a 4 or 12-week WD containing 21% fat and 0.15–0.2% cholesterol (Sniff diets). Tail samples were used for ACKR3 knockout genotyping in endothelial- and SMC-ACKR3-deficient mouse models with the following primers: forward primer: 5’ GAG TCA ATT GAG TGG GCA AGG 3’ and reverse primer: 5’ GCT ACA TTG CTT TCT TGA AGA AACC 3’ (Supp. Fig. S1A, B). For the bone marrow transplantation study (see details below), *Cre*^*ERT2*^*Ackr3*^*fl/fl*^*Apoe*^*−/−*^ mice (expressing the *Cre* ubiquitously) were used as donor mice, whereas *Apoe*^*−/−*^ mice were used as recipient mice. *Cre*^*ERT2*^*Ackr3*^*fl/fl*^*Apoe*^*−/−*^ mice were generated as follows: *Cre*^*ERT2*^ (Gt(ROSA)26) mice were purchased from Taconic Laboratories and bred with *Ackr3*^*fl/fl*^*Apoe*^*−/−*^ mice. In these mice, ACKR3 knockout genotyping was performed in spleen samples due to high blood content in this tissue (Supp. Fig. S1C). All mice were on a C57BL/6 J background. All animals were bred and housed in the local animal facility under specific pathogen free (SPF) conditions. Prior to the start of the WD, all mice were fed a normal chow diet. All animal experiments were approved by the local ethical committee (Regierung von Oberbayern, Sachgebiet 54, Germany; Az. 55.2.1.54-2532-177-2016 and ROB-55.2-2532.Vet_02-18-96).

### Bone marrow transplantation

All mice were irradiated twice in an X-ray machine (FAXITRON CP-160) with the program as follows: 5 Gy; kV160; mA 6.3; time 8.5; level 7. Mice were, after irradiation, treated with 1:1 neomycin (100 mg/ml in sterile dH2O) and polymyxin (0.1 g/ml in sterile dH2O) antibiotic solution for 4 weeks in their drinking water. After the irradiation, recipient *Apoe*^*−/−*^ mice were intravenously injected with bone marrow cells collected from *Cre*^*ERT2−*^*Ackr3*^*fl/fl*^*Apoe*^*−/−*^ and *Cre*^*ERT2*+^*Ackr3*^*fl/fl*^*Apoe*^*−/−*^ mice. Bone marrow cells from donor mice were prepared as follows: femurs and tibia of donor mice were collected without fractures and the bones were sterilized in 70% ethanol for 30 s and washed in PBS. The marrow of the bones was extracted under sterile conditions and a single cell suspension was prepared in sterile PBS by filtering the bone marrow with a 70 µm cell strainer. Syringes were prepared with 100uL of cell suspension (2.5 × 10^6^ cells per recipient mouse). After 4 weeks of recovery time, mice were treated with tamoxifen (1.5 mg per 20 g body weight, dissolved in corn oil) for 5 consecutive days. Mice were then subjected to 12 weeks of WD containing 21% fat and 0.15–0.2% cholesterol (Sniff diets).

### Endothelial ACKR3 imaging

Atherosclerotic plaque samples obtained during carotid endarterectomy were collected in the Maastricht pathology tissue collection (MPTC) in line with the Dutch code for proper secondary use of human tissue (http://www.fmwv.nl) and the local Medical Ethical Committee (protocol number 16-4-181). This study conforms to the Declaration of Helsinki, all participants have given informed written consent prior to the inclusion. Sections were stained immunohistochemically with von Willebrand factor (Abcam) and CXCR7 (ACKR3) (ThermoFisher) antibodies.

### Lesion analysis

Atherosclerotic lesion sizes were examined in plaque-prone areas such as the aortic root, arch and the aorta. Heart samples were fixed in 4% PFA and embedded in Tissue-Tek O.C.T. compound (Sakura) for cryo-sectioning (4 µm). Aortic arch samples were fixed likewise and embedded in paraffin for sectioning (4 µm). Aortic root and arch slides were stained with hematoxylin and eosin for lesion size assessments on ImageJ Software (three sections per mouse). Aortas were prepared en face and stained with Oil-Red-O for the quantification of lipid-laden lesion sizes on ImageJ Software. Plaque stability was examined in the Masson’s trichrome-stained aortic roots via Leica Software. Cellular composition of the lesions (macrophages, SMCs) as well as adhesion molecules (ICAM-1, 3E2, BD Biosciences) were studied via immunohistochemical stainings on the aortic root samples. Macrophages were identified with anti Mac-2 (M3/38, Cedarlane) antibody, SMCs were identified with anti-SMC (1A4, Dako) antibody and nuclei were stained with Hoechst (Invitrogen) solution (1:2000 in PBS) for 5 min or labeled with ProLong™ Diamond Antifade Mountant with DAPI (Thermofisher). NF-kB expression in atherosclerotic endothelial cells was assessed via immunohistochemical stainings on the aortic root samples with van Willebrand factor (vWF) (Abcam) and phospho-NF-kB p65 (SantaCruz) antibodies.

### Ex vivo perfusion assay

Leukocyte adhesion onto the ACKR3-deficient endothelium was analyzed by ex vivo perfusion of fluorescently labeled leukocytes on carotid arteries collected from control and *BmxCre* mice employing 2-photon excitation microscopy as described [58]. Isolated carotid arteries are mounted in vessel chambers and isolated and cell tracker green-labelled bone-marrow derived leukocytes are perfused due to the vessel. After washing, mounted carotid arteries were visualized using a LeicaSP5IIMP two-photon laser scanning microscope with a Ti: sapphire laser (Spectra Physics MaiTai Deepsee) tuned at 800 nm and a 20 × NA1.00 (Leica) water dipping objective. Spectral detection was performed using Hybrid Diode detectors (*n* = 4) tuned for maximum intensity of the (auto) fluorescence signal of the arterial wall and clear detection of cell tracker green: second harmonics generation of collagen (395–405 nm), autofluorescence; (470–490 nm), cell tracker green + autofluorescence; (500–550 nm), autofluroescence (570–600 nm). Adhered celltracker-positive leukocytes were counted manually by three observers in 3D sections of the arterial wall. 3D image processing was performed using LASX software including 3D analysis plugin (Leica).

### Intravital microscopy

Intravital microscopy was performed in the bifurcation of the left carotid artery by means of epifluorescence microscopy. The right jugular vein was canulated with a catheter for antibody and dye injection. After exposure of the left carotid artery, antibodies (1 μg) to CD11b (M1/70, Biolegend), Ly6C (HK1.4, Biolegend) and Ly6G (1A8, Biolegend) were sequentially administered to label myeloid cells, neutrophils and classical monocytes, respectively. Recordings were made 3 min after injection of each antibody. The diameters of the external carotid arteries were 268.8 ± 34.4 µm in the control group and 267.4 ± 37.1 µm in the endothelial ACKR3 deficient group. Intravital microscopy was performed using an Olympus BX51 microscope equipped with a Hamamatsu 9100-02 EMCCD camera and a ×10 saline-immersion objective. For image acquisition and analysis, Olympus Cell-R software was used.

### Leukocyte infiltration

Blood leukocyte tracking was performed according to the protocol described in Winter et al*.* [61]. Briefly, mice were injected with a rat anti-CD45 antibody (30-F11, BioLegend) and killed 2 h post-injection. Cryo-sectioned aortic root samples from the mice were then stained with an anti-rat secondary antibody to trace back the pre-labelled circulating immune cells which infiltrated into the aortic root lesions in the 2-h timeframe.

### In vivo endothelial permeability assay

0.5% Evans blue (EVB) was prepared in 0.9% saline solution and the solution was filter-sterilized. Mice were injected with the EVB solution 40 mg/kg and sacrificed 30 min post-injection. Mice were then perfused with sufficient phosphate-buffered saline (PBS) and following organs were collected to analyze the retained amount of EVB: lung, aorta, aortic arch. Aortic arches were fixed with 4% paraformaldehyde (PFA) for 30 min and placed on objective slides to be imaged (EVB excitation peaks: 470 and 540 nm, emission peak at 680 nm). Tilescan z-stacks of the whole aortic arches were taken with a Leica SP8 3X confocal microscope tube for efficient EVB detection and processed using Imaris 8.4 (Oxford instruments). The endothelial Evans blue–positive signal was quantified as a volume after 3D segmentation based on appropriate fluorescence intensity thresholding. Aortas and lungs were weighed, air dried for half a day, fixed with formamide and cut into small pieces. The organs were then incubated at 56 °C shaking for 24 h to release the retained EVB. Optical density (OD) of the formamide solutions containing the EVB was then measured at 620 nm and normalized to tissue weights.

### Lipid measurement

Cholesterol and triglyceride levels were quantified in EDTA-plasma samples using enzymatic assays (c.f.a.s. cobas, Roche Diagnostics) according to the manufacturer’s protocol.

## ELISA

CXCL12 levels in EDTA-plasma samples were measured via commercially available Mouse CXCL12/SDF-1 alpha Quantikine ELISA Kit (R&D Systems), according to the manufacturer’s instructions. Phosphorylation of p65 subunit NFkB was quantified via NF kappaB p65 (pS536 + Total) ELISA Kit (Abcam), according to the manufacturer’s instructions.

### Cell culture

Human Primary Coronary Artery Endothelial Cells (CC-2585), EGM-2 Bulletkit medium (CC-3162) and ReagentPack Subculture Reagents (CC-5034) were purchased from Lonza. The complete medium contained the following: 2% FBS, 0.2 mL hydrocortisone, 2 mL hFGF-B, 0.5 mL VEGF, 0.5 mL R3-IGF-1, 0.5 mL ascorbic acid, 0.5 mL hEGF, 0.5 mL hEGF, 0.5 mL GA-1000 as well as 5 mL penicillin–streptomycin antibiotic mix. Cells were kept at 37 °C and 5% CO_2_ under sterile conditions in a humidified incubator. Subculturing and medium refreshing were performed according to the manufacturer’s protocol. For ACKR3 silencing studies, cells were transfected with either 30 nM negative control siRNA (Silencer™ Negative Control No. 1 siRNA, AM4611 ThermoFisher) or 30 nM ACKR3 siRNA (Silencer validated siRNA CXCR7, AM51331 Ambion by Life Technologies) with the following sequence: Sense GGAZGACACUAAUUGUUAGtt and Antisense CUAACAAUUAGUGUCAUCCtt. Transfection was performed via siPORT™ NeoFX™ Transfection Agent according to the manufacturer’s protocol (Invitrogen by Life Technologies). Cells were transfected for 48 h and the transfection efficiency was evaluated by droplet digital PCR. Cells were treated with 10 ng/mL TNFα 2–4 h before evaluating the impact of ACKR3 silencing. ACKR3-transfected HEK cells were kindly donated by Prof. Alexander Faussner and treated with tetracycline for 24 h to induce ACKR3 expression and with 10 ng/mL TNFα for 1 h before evaluating the impact of ACKR3 overexpression. NF-kB p65 phosphorylation was evaluated via ELISA (see above) on TNFα-stimulated ACKR3-induced HEK cells as well as on ERK inhibitor (SCH772984, Selleckchem, 2 µM) and Akt inhibitor (MK-2206-2HCl, Selleckchem, 20 µM) treated ACKR3 induced HEK cells. Static adhesion assay with THP-1 cells was performed as follows: HCAECs were transfected with 30 nM negative control siRNA or 30 nM ACKR3 siRNA for 48 h. In addition, negative control siRNA-transfected cells were also treated with ERK inhibitor (2 µM) and Akt inhibitor (10 µM) for 2 h prior to 10 ng/mL TNFα treatment for 2 h. THP-1 cells were stained with calcein for 30 min and then co-incubated with pre-treated HCAECs for 30 min after washing. HCAECs were then washed twice and the fluorescence measurement was performed at 485/535 nm wavelength with a TECAN infinite 200pro plate reader.

### RNA isolation

Total RNA isolation from cell culture and mouse aortic arch samples was performed by commercially available RNA isolation kit from Zymoresearch (Direct-zol microprep kit) according to the manufacturer’s protocol. The quality (A_260_/A_280_) and the quantity (ng/µL) of the RNA was measured by NanoPhotometer N60/N50 (Implen). A ratio of ~ 2 for A_260_/A_280_ was accepted as good quality RNA.

### cDNA synthesis

RNA samples were diluted to the same concentration and the cDNA synthesis was performed via the commercially available iScript cDNA synthesis kit from Bio-Rad according to the manufacturer’s protocol.

### Droplet digital PCR

PCR was performed on QX200 Droplet Digital PCR (ddPCR™) system from Bio-Rad. 20µL reaction mixes were prepared using 10μL of 2X ddPCR SuperMix for probes (No dUTP) (Bio-Rad), 1µL of 20X FAM-labeled primer/probe for the target gene, 1µL of 20X VIC-labeled primer/probe for the housekeeping gene, RNase-/DNase-free water and cDNA sample. Taqman primers were purchased from Thermofisher Scientific. Droplet generation was performed in the QX200 droplet generator (Bio-Rad) by adding 20µL reaction mix and 70µL droplet generation oil for probes (Bio-Rad) onto DG-8 cartridges covered with gaskets (Bio-Rad). 42µL of the droplet solution (containing up to 20,000 droplets) was transferred to the appropriate PCR plate (Bio-Rad) which was then sealed with a piercing foil using the PCR plate sealer (Bio-Rad). Cycling was performed in the ddPCR cycler with the following conditions: 10 min at 95 °C (enzyme activation), 30 s at 94 °C (denaturation) and 1 min at 60 °C (annealing/extension) for 40 cycles, 10 min at 98 °C (enzyme deactivation). The PCR plate was then proceeded to the droplet reader (Bio-Rad). Analysis was performed on QuantaSoft software (Bio-Rad).

### Flow cytometry

Whole blood was collected from mice in EDTA-buffer tubes and red blood cell lysis was performed prior to stainings. Hematopoietic cell populations in blood samples were stained as follows: anti-CD45 (30-F11, eBioscience), anti-CD115 (AFS98, eBioscience), anti-Gr1 (RB6-8C5, eBioscience), anti-CD11b (M1/70, eBioscience), anti-B220 (RA3-6B2, eBioscience) and anti-CD3 (145-2C11, eBioscience). Cell populations were gated and analyzed as follows using FlowJo Software: leukocytes (CD45^+^), neutrophils (CD45^+^CD115^−^Gr1^high^), monocytes (CD45^+^CD11b^+^CD115^+^), T cells (CD45^+^CD3^+^) and B cells (CD45^+^B220^+^). For cell culture experiments, the cells were washed and trypsinized at the end of the ACKR3 silencing treatments (72 h) to be collected as single cell suspensions. The samples were stained as follows: anti-ICAM (HA58, eBioscience), anti-VCAM (429, BD Biosciences) and anti-E-selectin (10E9.6, BD Biosciences). All samples were analyzed with FACS Canto II together with FACSDiva software (BD Biosciences).

### Protein isolation

Cell culture samples were lysed with M-PER™ Mammalian Protein Extraction Reagent (from ThermoFisher Scientific) according to the manufacturer’s protocol. Total protein levels in the lysates were measured via a commercially available BCA assay kit (Pierce™ BCA™ Protein-Assay from ThermoFisher Scientific) according to the manufacturer’s protocol.

### Phosphorylation array

Silenced and control cell culture samples were analyzed for the modifications in the MAPK pathway with the commercially available Human/Mouse MAPK Phosphorylation Array kit (AAH-MAPK-1-8) from RayBiotech, according to the manufacturer’s protocol.

### Western blot

Equal amounts of protein samples obtained from tissue homogenates were mixed with Novex™ NuPAGE™ LDS sample buffer (4X) and boiled at 95 °C for 5 min. Lysates were then loaded onto 12% Mini-PROTEAN^®^ TGX™ Precast Protein Gels (Bio-Rad) along with Precision Plus Protein™ WesternC™ blotting standard (Bio-Rad). Western blot was performed with Mini Trans-Blot^®^ Cell and Criterion™ Blotter according to the manufacturer’s protocol (Bio-Rad). Briefly, protein separation was achieved by gel electrophoresis using running buffer (Tris/glycine/SDS from Bio-Rad) for about 1 h with 100–150 Volts (V). Proteins were then transferred from the gel to the membrane in transfer buffer (Tris/glycine buffer from Bio-Rad with 20% methanol added) for 1 h at 100 V. The membrane was then blocked with 5% bovine serum albumin (BSA) in Tris-buffered saline with Tween 20 buffer (TBST from Bio-Rad) for 1 h. Primary antibody incubation against p-Erk1/2 (Abcam) and p-p65-NFkB (SantaCruz) was carried out overnight at 4 °C. The membrane was then washed with TBST buffer and the HRP-labelled anti-rabbit secondary antibody (from Abcam) incubation was carried out for 1 h at room temperature. The membrane was washed again with TBST buffer and developed with SuperSignal™ West Pico PLUS Chemiluminescent Substrate (from Thermofisher). After the signal for the phospho-proteins was captured, the membrane was again washed in TBST, stripped for 30 min with Restore™ Western Blot Stripping Buffer (from Thermofisher) and blocked for 1 h with 5% BSA solution. The membrane was then incubated with antibodies against t-Erk1/2 (Abcam) and t-p65-NFkB (SantaCruz) for 1 h at room temperature and this was followed by again 1-h incubation of secondary antibody. The signal was captured as explained above. Analysis of phospho- and total protein signals was performed on ImageJ and the phospho-protein signal was normalized by dividing it to total protein signal.

### Quantification and statistical analysis

All data are expressed as mean ± SEM. Statistical analyses were performed using GraphPad Prism version 7.0 or higher (GraphPad Software, Inc.). Outliers were identified with ROUT = 1 and normality of the data was tested via the D’Agostino–Pearson omnibus normality test. Statistical significance was tested via unpaired Student’s *t* test with Welch correction for normally distributed data and Mann–Whitney *U* test for non-normally distributed data. A result of < 0.05 for *p* value was considered statistically significant. *p < 0.05, **p < 0.01, ***p < 0.001.

## Results

### ***Endothelial ACKR3 deficiency protects against atherosclerosis in Apoe***^***−/−***^*** mice***

**Fig. 1:**
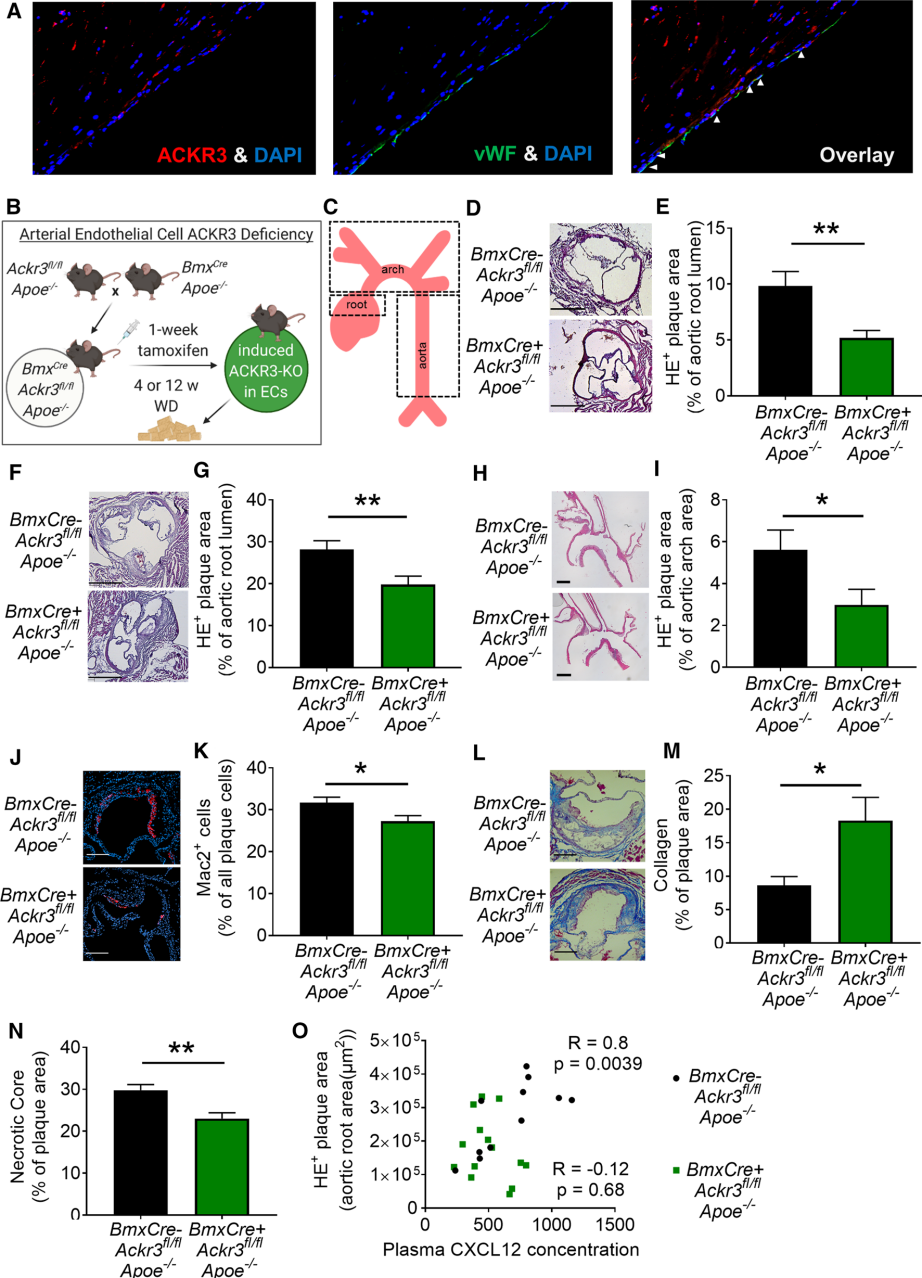
Endothelial ACKR3 deficiency attenuates atherosclerosis in hyperlipidemic mice. **A** Expression of endothelial ACKR3 in human carotid artery atherosclerotic lesions. **B** Schematic representation of the 4-week WD experimental setup (created with BioRender.com). **C** Schematic representation of the studied atherosclerosis prone regions. **D** Representative images (scale bar: 500 µm) and **E** quantification of atherosclerotic lesion sizes in the aortic roots of mice fed with 4 weeks of WD (*n* = 22–32). **F** Representative images (scale bar: 500 µm) and **G** quantification of atherosclerotic lesion sizes in the aortic roots of mice fed 12 weeks of WD (*n* = 12–14). **H** Representative images (scale bar: 500 µm) and **I** quantification of atherosclerotic lesion sizes in the aortic arches (*n* = 12–13). **J** Representative images (scale bar: 250 µm) and **K** quantification of macrophage (MAC2 +) content in the aortic roots (*n* = 12). **L** Representative images (scale bar: 250 µm) and **M** quantification of collagen (*n* = 12–14) and **N** necrotic core content in the aortic roots (*n* = 12–13). **O** Spearman r correlation of plasma CXCL12 levels and aortic root lesion content in mice fed 12 weeks of WD (*R* = 0.8, p = 0.0039), (*n* = 12–14). Results represent Mean ± SEM. *p < 0.05, **p < 0.01

Vascular cells, more specifically endothelial (EC) and smooth muscle (SMC) cells are described to express ACKR3 [5, 25]. Furthermore, we could also reveal ACKR3 expression in ECs of human atherosclerotic lesions (Fig. 1A). To study the role of vascular ACKR3 in atherosclerosis we crossbred *Ackr3*^*fl/fl*^ mice with *BmxCreER*^*T2*^ (arterial EC specific) and *SmmhcCreER*^*T2*^ (SMC specific) expressing mice. To establish hematopoietic ACKR3 deficient animals (htACKR3) we transplanted bone marrow from animals with a systemic ACKR3 deficiency (UniCre^ERT2^ *Ackr3*^*fl/fl*^) into *Apoe*^*−/−*^ mice. All mice used were *Apoe*^*−/−*^ background. Mice lacking endothelial ACKR3 (EC-ACKR3) were subjected to 4-week and 12-week WD in order to evaluate early onset and advanced atherosclerotic lesion formations (aortic root and arch), respectively (Fig. 1B, C). Analyses revealed that EC-ACKR3 deficiency significantly decreased lesion sizes in the aortic roots of mice after 4 weeks of WD (Fig. 1D, E) and significantly reduced lesion sizes in both aortic roots and arches after 12 weeks of WD (Fig. 1F–I). In SMC-specific and hematopoietic ACKR3 deficient mice (after 12 weeks WD), however, we observed no differences in atherosclerotic lesion sizes in the aortic roots, arches and the aorta (Supp. Fig. S2A-N). Further analyses of the advanced lesions disclosed that EC-ACKR3 deficient mice had significantly less macrophages within their lesions (Fig. 1J, K) while the macrophage content of SMC-specific and hematopoietic ACKR3 deficient mice remained unchanged (Supp. Fig. S3A-C). SMC content was unaffected in all three models (Supp. Fig. S3D-F), whereas lesional collagen content was significantly increased in SMC-ACKR3-deficient (Supp. Fig. S3G) and EC-ACKR3-deficient (Fig. 1L, M) lesions but not in hematopoietic ACKR3-deficient lesions (Supp. Fig. S3 H, I). Necrotic core size in EC-ACKR3-deficient lesions was reduced (Fig. 1N). Of note, lesional SMC content correlated significantly with the collagen density (Supp. Fig. S4A), suggesting that SMCs may be responsible for the enhanced plaque stability through collagen production. Plasma lipid levels did not differ between control and EC-ACKR3 mice (Table 1) as well as in control, SMC-ACKR3 and hematopoietic ACKR3 deficient mice (data not shown). Interestingly, mice lacking EC-ACKR3 developed leukocytosis (Table 1). While CXCL12 levels did not differ in the plasma (Supp. Fig. S4B) between control and EC-ACKR3-deficient mice, circulating CXCL12 levels correlated significantly with atherosclerotic lesions sizes in the control mice, whereas this correlation was lost in the absence of endothelial ACKR3, suggesting that ACKR3–CXCL12 interactions drive atherosclerosis (Fig. 1O). Hence, EC-specific loss of ACKR3 clearly protects against atherosclerosis under hyperlipidemic conditions.

**Table 1:** General characteristics of mice

12 weeks WD	*BmxCre-* *Ackr3* ^*fl/fl*^ *Apoe*^*−/−*^	*BmxCre* + *Ackr3*^*fl/fl*^ *Apoe*^*−/−*^	*P* value
Leukocytes [× 10^6^/ml]	1.3 ± 0.2	2.1 ± 0.2	0.011*
Neutrophils [× 10^5^/ml]	3.5 ± 0.8	6.4 ± 0.8	0.017*
Classical monocytes [× 10^5^/ml]	1.0 ± 0.2	1.2 ± 0.1	0.541
Non-classical monocytes [× 10^4^/ml]	8.9 ± 1.9	8.2 ± 1.5	0.776
B cells [× 10^5^/ml]	5.0 ± 1.0	8.2 ± 0.9	0.022*
T cells [× 10^5^/ml]	1.8 ± 0.3	2.6 ± 0.3	0.072
Thrombocytes [× 10^3^/µl]	1118 ± 162	977 ± 146	0.523
Cholesterol [mg/dL]	1208.0 ± 100.9	984.8 ± 144.4	0.219
Triglycerides [mg/dL]	181.4 ± 17.8	132.2 ± 26.0	0.133
Bodyweight [g]	30.7 ± 2.4	33.6 ± 1.7	0.338

Bold italic values are *p* < 0.05

Circulating leukocyte and leukocyte subset numbers quantified by flow cytometry, plasma cholesterol and triglyceride levels, as well as body weight of 12-week WD fed mice. Results represent mean ± SEM

^*^p < 0.05

### Endothelial ACKR3 deficiency reduces immune cell infiltration into lesions without affecting vascular integrity

**Fig. 2:**
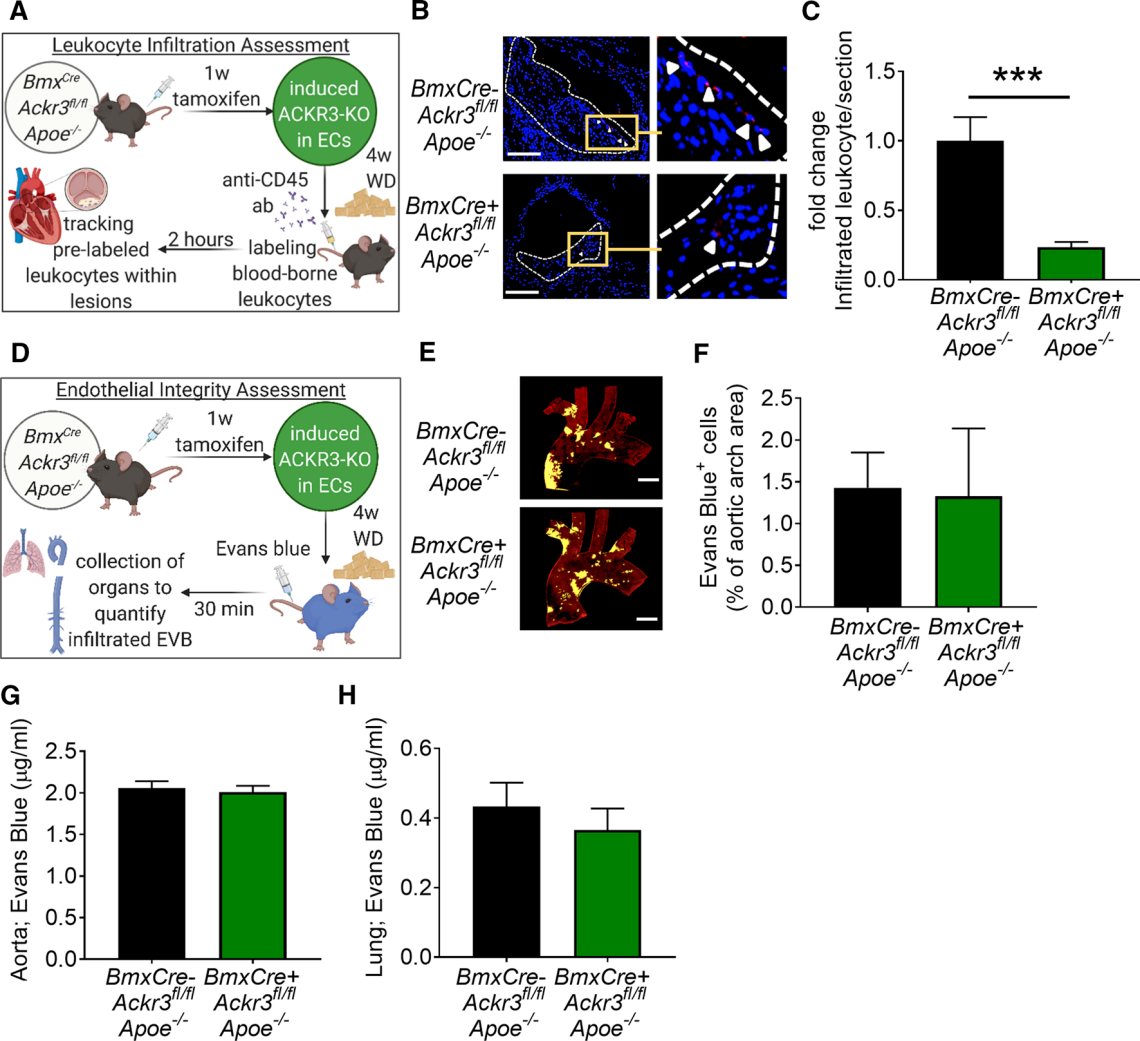
Endothelial ACKR3 deficiency decreases leukocyte infiltration into atherosclerotic lesions without affecting endothelial permeability. **A** Schematic representation of the leukocyte tracking experimental setup (created with BioRender.com). **B** Representative images (scale bar: 500 µm) and **C** quantification of infiltrated leukocytes in aortic root lesions (*n* = 7–10). **D** Schematic representation of the endothelial permeability experiment. **E** Representative images and **F** quantification of infiltrated EVB in the aortic arches (*n* = 4–5). **G** Quantification of infiltrated EVB in the aortas (*n* = 7) and **H** lungs (*n* = 6). Results represent Mean ± SEM. ***p < 0.001

Since our experimental model involves modification of the vascular endothelium through a receptor knockout, we hypothesized that endothelial ACKR3 may be involved in leukocyte extravasation, which may explain the changes in the lesional macrophage content between control and EC-ACKR3-deficient mice. To test if leukocyte infiltration into the lesions is affected by EC-ACKR3 deficiency, we followed the protocol described in Winter et al*.* [61]. Briefly, 4-week WD-fed control and EC-ACKR3-deficient mice were injected intravenously with an anti-CD45 antibody and the presence of this antibody was tracked in the atherosclerotic lesions as an indicator of leukocyte infiltration (Fig. 2A). We observed significantly less infiltrated leukocytes in the lesions of EC-ACKR3-deficient mice (Fig. 2B, C), suggesting that endothelial ACKR3 is involved in the regulation of leukocyte extravasation. Following these findings, we hypothesized that EC-ACKR3 may mediate leukocyte infiltration by affecting endothelial permeability. The permeability of the endothelial barrier is a critical factor impacting the trafficking of blood-borne elements into the sub-endothelial space. To investigate whether EC-ACKR3 deficiency leads to ‘leakiness’ of the vascular endothelium, we injected EC-ACKR3-deficient and control animals with EVB and quantified the amount of EVB that penetrated through the endothelium (Fig. 2D). No significant differences were observed in EVB quantities in the aortic arches (Fig. 2E, F), aortas (Fig. 2G) or in the lungs (Fig. 2H) of the control and EC-ACKR3 deficient mice, demonstrating that EC-ACKR3 does not affect vascular integrity.

### Endothelial ACKR3 deficiency attenuates endothelial adhesion

**Fig. 3:**
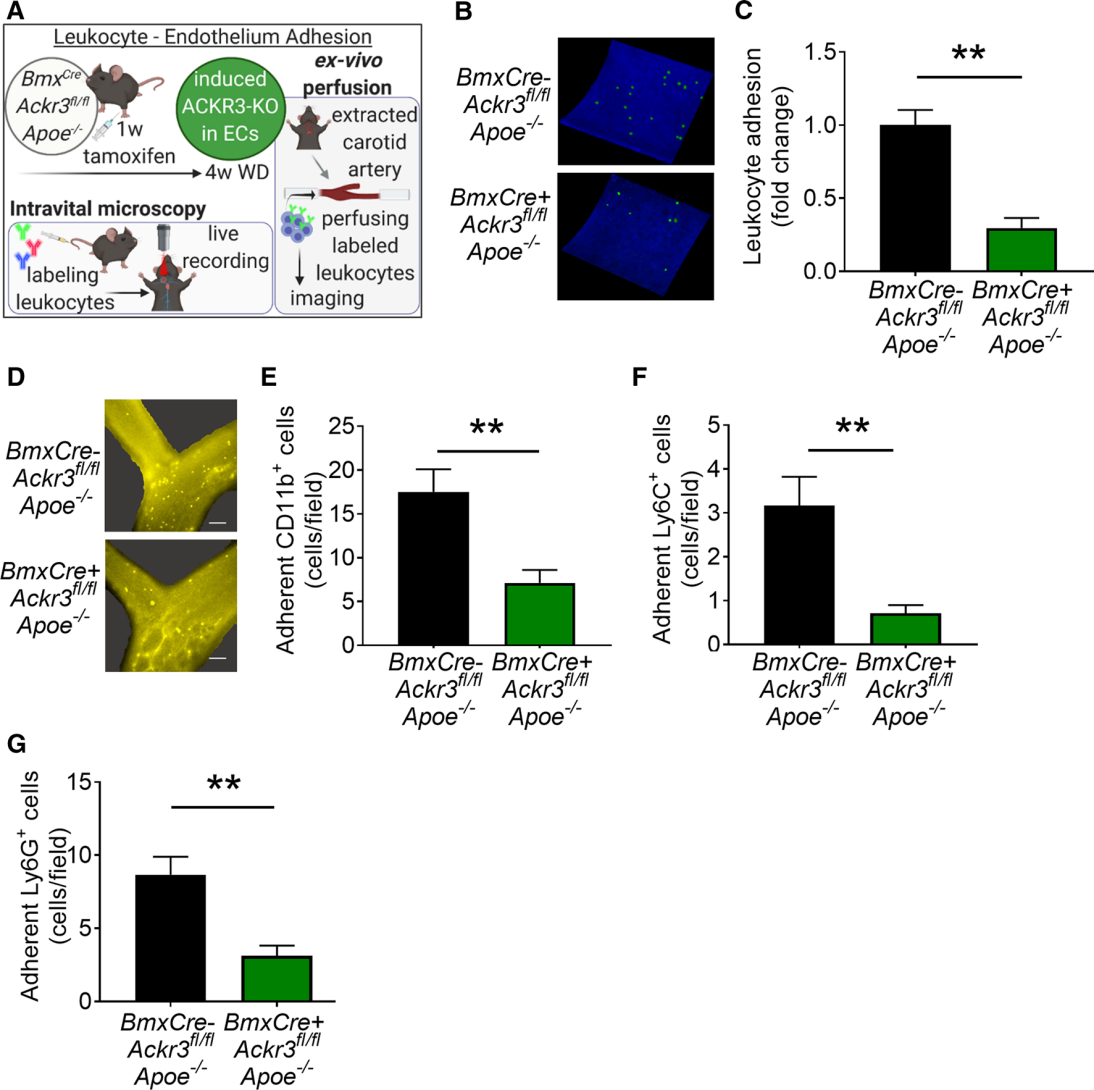
Endothelial ACKR3 deficiency decreases endothelium-immune cell adhesion in arteries. **A** Schematic representation of the ex vivo perfusion and intravital microscopy experimental setup (created with BioRender.com). **B** Representative images and **C** fold change of adhered leukocytes onto ex vivo perfused carotid arteries (*n* = 5). **D** Representative images of intravital microscopy (scale bar: 100 µm) and quantification of adherent **E** CD11b + , **F** Ly6C + and **G** Ly6G + cells (*n* = 6–8). Results represent Mean ± SEM. **p < 0.01

Next, the potential impact of EC-ACKR3 on leukocyte–endothelium adhesion was tested to elucidate EC-ACKR3-mediated decrease in arterial immune cell invasion. To this end, we performed an ex vivo arterial perfusion assay (Fig. 3A). Viable carotid arteries from control and EC-ACKR3-deficient mice were collected and perfused ex vivo with fluorescently labeled leukocytes. Leukocyte adhesion to arteries with endothelial ACKR3 deficiency was markedly decreased suggesting that ACKR3 is involved in the regulation of the endothelial adhesion processes (Fig. 3B, C). To further confirm our findings, we tested the impact of endothelial ACKR3 on vascular adhesion in vivo via intravital microscopy (Fig. 3D). Indeed, deficiency of EC-ACKR3 strongly limited the adhesion of leukocytes, which was tested in different subsets: myeloid cells (Fig. 3E), classical monocytes (Fig. 3F) and neutrophils (Fig. 3G) were investigated individually and all subsets were observed to adhere significantly less to the arteries of the EC-ACKR3 deficient mice.

### ACKR3 silencing decreases adhesion molecule expression in mouse lesions and human coronary artery endothelial cells

**Fig. 4:**
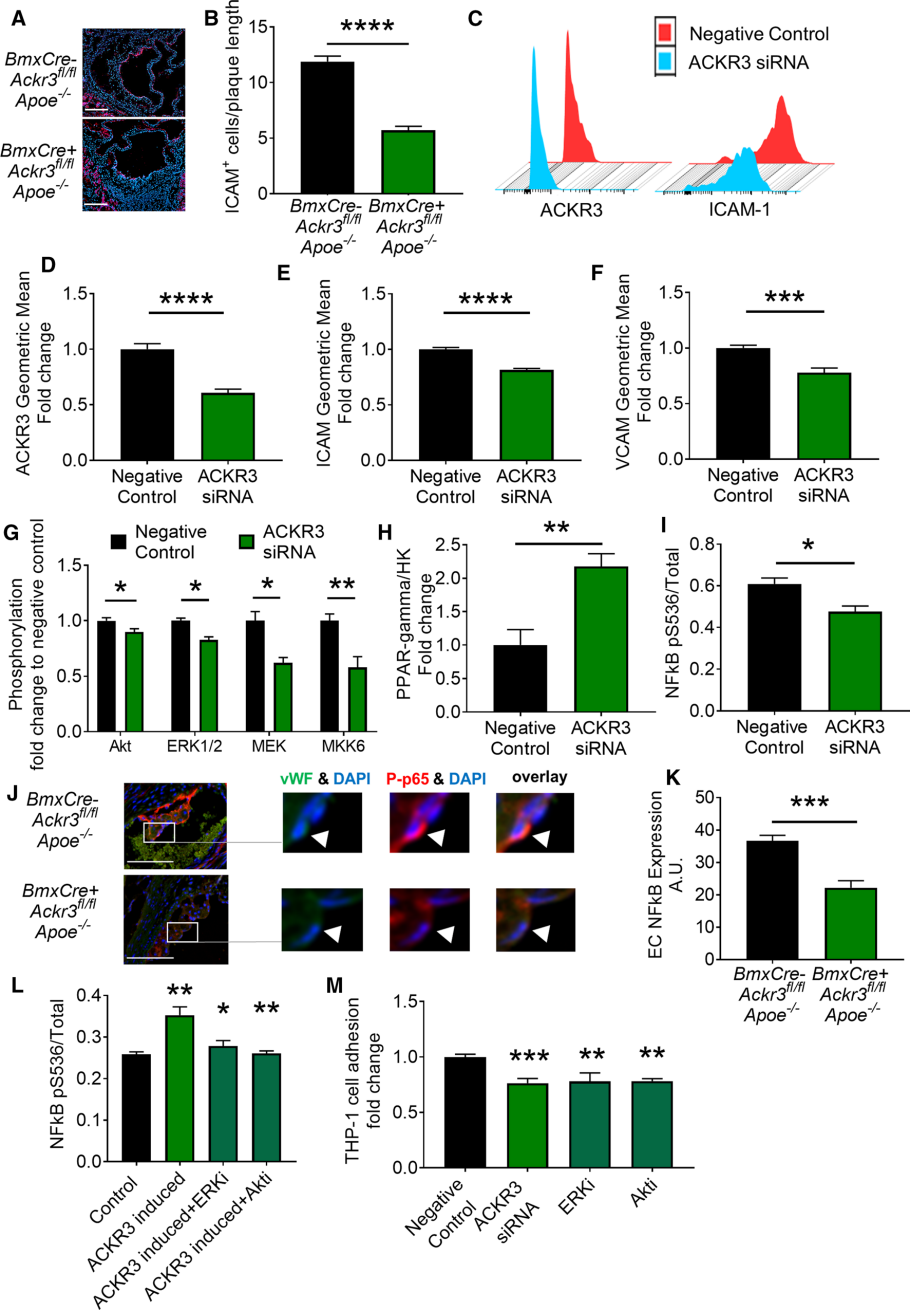
ACKR3 silencing decreases endothelial adhesion molecule expression along with decreased MAPK and NF-kB signaling in human coronary artery endothelial cells. **A** Representative images (scale bar: 250 µm) and **B** quantification of ICAM + cells on the endothelial lining of mouse aortic root lesions (*n* = 12–14). **C** Representative flow cytometry histograms of ACKR3 and ICAM-1 expression (geometric mean by flow cytometry) of **D** ACKR3, **E** ICAM, and **F** VCAM in control and silenced HCAECs stimulated with TNF-α (*n* = 3 independent batches). **G** Quantification of protein phosphorylation in the MAPK pathway (*n* = 4) in HCAECs stimulated with TNF-α. **H** Expression of *PPAR-γ* measured by ddPCR in control and silenced cells (*n* = 8–9) stimulated with TNF-α. **I** Phosphorylation of the p-65 NF-kB subunit quantified by ELISA in control and *ACKR3*-silenced HCAECs stimulated with TNF-α (*n* = 3). **J** Representative images (*green*  von Willebrand factor, *red*  ACKR3, *blue*  DAPI) (scale bar: 100 µm) and **K** Quantification of phospho-p65 NF-kB expression in aortic root endothelial cells from control and knockout mice. A.U. represents arbitrary units (*n* = 3). **L** Phosphorylation of the p-65 NF-kB subunit quantified by ELISA in control and ACKR3-induced as well as ERK (SCH772984) and Akt inhibitor (MK-2206-2HCl)-treated HEK cells (*n* = 3) stimulated with TNF-α. **M** Adhesion of THP-1 cells onto HCAECS treated with ACKR3 siRNA as well as ERK (SCH772984) and Akt (MK-2206-2HCl) inhibitors (*n* = 7–12). Results represent Mean ± SEM. *p < 0.05, **p < 0.01, ***p < 0.001, ****p < 0.0001

**Fig. 5:**
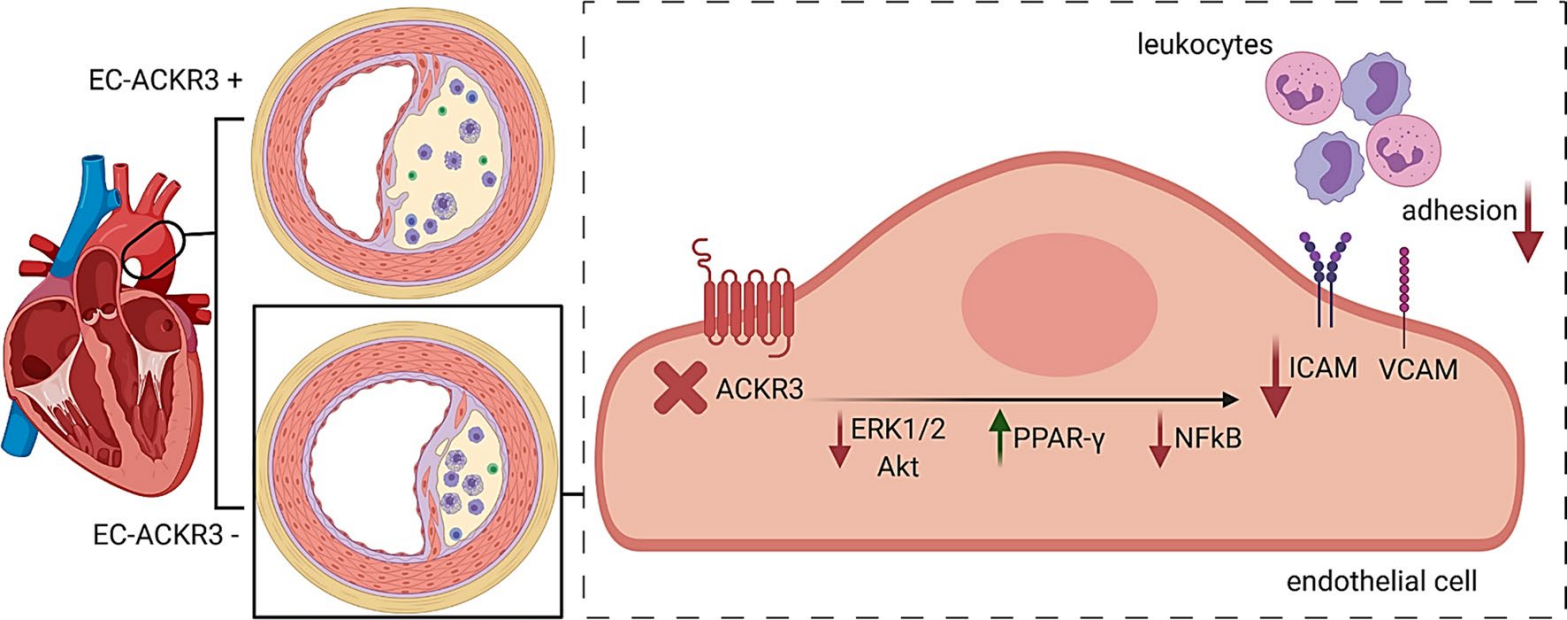
Summary of endothelial ACKR3 in atherosclerosis. Graphical summary of EC-ACKR3-mediated processes (created with BioRender.com). Deficiency of ACKR3 in arterial endothelial cells leads to decreased atherosclerotic lesion sizes concomitant with decreased endothelial-immune cell adhesion. Endothelial ACKR3 silencing leads to downregulation of adhesion molecules and phosphorylated ERK1/2, Akt and NF-kB p65, which are inflammatory pathways involved in cell adhesion. Meanwhile, *PPAR-γ*, which suppresses NF-kB, is upregulated

In order to understand how EC-ACKR3 affected endothelial adhesion in mice, we examined adhesion molecule expression in atherosclerotic lesions by immunohistochemistry and observed significantly less intracellular adhesion molecule-1-positive (ICAM-1 +) cells in the aortic root lesions of EC-ACKR3-deficient mice (Fig. 4A, B). As ACKR3 is also expressed in human atherosclerotic endothelium (Fig. 1A), we sought to investigate whether ACKR3 can regulate adhesion molecule expression in human endothelial cells as well. In order to study this potential human link, we silenced *ACKR3* in human coronary artery endothelial cells (HCAECs) (Fig. 4C, D; Supp. Fig. S5A, B) and investigated its impact on adhesion molecule expression upon TNF-α stimulation. Flow cytometry analysis revealed that silenced HCAECs express significantly less ICAM (Fig. 4C, E) and vascular cell adhesion molecule VCAM (Fig. 4F) compared to control samples. In line with this finding, expression of these adhesion molecules correlated significantly with the expression of ACKR3 in the HCAECs (Supp. Fig. S5C, D). Although ACKR3 has long been regarded as a non-signaling receptor [6, 40, 57, 60], further research in recent years revealed that ACKR3 can signal through β-arrestin and mitogen activated protein kinase (MAPK) pathways, as well as NF-kB [1, 27, 31, 33, 38, 41, 42, 67]. In order to investigate whether the ACKR3 deficiency dependent decrease in adhesion molecule expression could be due to the downregulation of these pathways, we performed a MAPK phosphorylation antibody array on *ACKR3* silenced and control HCAECs. *ACKR3* silencing revealed significant downregulation of the key mediators in the MAPK pathway including decreased ERK1/2 phosphorylation (Fig. 4G). We further confirmed reduced ERK1/2 phosphorylation in silenced cells via western blot (Supp. Fig. S5E). Moreover, we and Li et al*.* have previously shown that ACKR3 can regulate PPAR-γ expression in adipose tissue [18, 32]. Here we show that this is also true for HCAECs as ACKR3 silenced cells showed increased expression of the anti-inflammatory *PPAR-γ* (Fig. 4H), which has been established to inhibit the expression of ICAM and VCAM when activated [43, 49]. Furthermore, PPAR-γ is known to be a negative regulator of NF-κB [45, 50, 54], which induces the transcription of ICAM and VCAM [29, 52]. In line with this, decreased phosphorylation of the NF-κB p-65 subunit in ACKR3 silenced HCAECs was confirmed via quantitative ELISA analysis (Fig. 4I) as well as western blot (Supp. Fig. S5F). To further confirm these findings in our mouse model, we quantified phospho-NF-kB p-65 expression in atherosclerotic endothelial cells via immunohistochemically stained aortic root lesions of 4-week WD fed mice (Fig. 4J). Our results indicated that EC-ACKR3 deficient mice had significantly less expression of phospho-NF-kB p-65 in their atherosclerotic endothelial cells (vWF +) (Fig. 4K), suggesting that ACKR3 modulates cell adhesion to endothelial cells most likely through NF-kB activation. The impact of ACKR3 on NF-κB activation was also confirmed with ACKR3-transfected HEK cells (Supp. Fig. S5G); cells with induced ACKR3 expression showed significantly higher NF-κB p-65 subunit phosphorylation compared to control cells, whereas the NF-κB phosphorylation was dampened by ERK and Akt inhibitors (Fig. 4L). These results suggest that ACKR3 activates NF-κB through ERK and Akt. The functional role of ERK and Akt was further validated using an adhesion assay in which inhibition of both ERK and Akt dampened THP-1 cell adhesion onto HCAECs in a similar manner as ACKR3 silencing (Fig. 4M). The effects of EC-ACKR3 on ECs in the context of atherosclerosis are summarized in Fig. 5.

## Discussion

Our study shows that arterial endothelial deficiency of ACKR3 in hyperlipidemic mice results in attenuated atherosclerosis, characterized by reduced lesion sizes in early and late stage atherosclerosis, decreased lesional macrophage and necrotic core content in addition to increased plaque stability. These findings establish a pro-atherosclerotic role of endothelial ACKR3 under hyperlipidemic conditions.

Arterial invasion of immune cells is a key atherosclerotic event endorsing sub-endothelial foam cell accumulation in arteries, which in turn fuels lesional growth. Our results indicated that endothelial deficiency of ACKR3 impaired leukocyte infiltration into the lesions of EC-ACKR3-deficient mice. It is not clear why ACKR3 deficiency in ECs lead to leukocytosis in mice and this remains to be elucidated in further studies. The fact that EC-ACKR3 deficiency hindered leukocyte entry into the lesions despite leukocytosis reflects that its effects are quite strong. Trans-endothelial migration of immune cells is a crucial step enabling arterial invasion and accumulation of the immune cells in the lesions. Our results showed that EC-ACKR3 regulated a main step in trans-endothelial migration of leukocytes, namely adhesion. Previous studies disclosed the role of ACKR3 in cell adhesion via in vitro functional assays using endothelial progenitor cells as well as cancer cells [11, 21, 37, 68]. Nonetheless, this role of ACKR3 has never been shown in vascular endothelium in vivo before, especially in the context of atherosclerosis. This finding concludes that the deficiency of endothelial ACKR3 partially protects hyperlipidemic mice from atherosclerosis by decreasing endothelial adhesion and thereby limiting immune cell migration into the lesions. A possible similar role of the hematopoietic ACKR3 does not seem likely as deficiency of ACKR3 in the hematopoietic compartment did not change lesional macrophage content and atherosclerotic lesion sizes in this study. Nevertheless, cell-specific ACKR3 studies within the hematopoietic compartment may still be evaluated for possible roles of ACKR3 on the immune cell subsets. For example, a recent study by Rohlfing et al. demonstrated that megakaryocyte/platelet-specific deletion of ACKR3 resulted in increased myocardial injury and inflammation following ischemia/reperfusion [47]. Furthermore, the loss of platelet ACKR3 enhanced platelet activation and thrombosis [47]. This study further emphasizes the need to further explore the role of hematopoietic cell-specific AKCR3 in more detail.

Based on the literature, ACKR3 seems to be an important receptor for immune cell infiltration in other disease models as well. For example, in an allergic airway inflammation mouse model, ACKR3 knockdown decreased immune cell infiltration into the lungs [7]. In an acute peritoneal inflammation model, ACKR3 inhibition reduced accumulation of polymorphonuclear granulocytes in the peritoneal fluid of mice along with a reduction in neutrophil infiltration into lungs and livers of the mice [41]. Even more interestingly, a mouse model of multiple sclerosis showed that endothelial expression of ACKR3 at the central nervous system (CNS) vasculature was increased during inflammation and the antagonism of ACKR3 dampened leukocyte infiltration into the CNS parenchyma resulting in improved the clinical severity of the disease model [10]. In addition to inflammatory models, the impact of ACKR3 on trans-endothelial migration is reported to be important in cancer studies as well, as this process allows malignant cells to metastasize [15]. Altogether, these findings establish a significant role of ACKR3 in the regulation of immune cell extravasation.

Previous studies investigating ACKR3 in zebrafish disclosed its ability to scavenge its ligands CXCL11 and CXCL12 [6, 40]. Of note, C57BL/6 (B6) mice do not express the chemokine CXCL11 [53], which is a limitation of this study as our results cannot account for possible differences of ACKR3 mediated atherosclerotic processes in the presence of CXCL11. Apart from CXCL11 and CXCL12, ACKR3 has several other ligands, such as macrophage inhibitory factor (MIF), adrenomedullin (ADM) and bovine adrenal medulla 22 (BAM22) [59]. These ligands also bind several other receptors, for example CXCL12 also binds CXCR4, and MIF also binds CXCR4, CD74 and CXCR2 [59]. Therefore, ACKR3 ligand treatments in cells may introduce non-ACKR3 dependent effects via several other receptors. In our in vitro studies, we focused exclusively on ACKR3 dependent effects by silencing the receptor in inflamed endothelial cells to mimic atherosclerotic inflammatory conditions. To this end, we treated the endothelial cells with a key inflammatory factor, TNF-α, which has also been established to induce ACKR3 [35]. It is also important to highlight the fact that ACKR3 has been shown to exert ligand-independent effects (internalization of the receptor), as well [36]. We did not observe any differences in circulating CXCL12 levels in mice lacking ACKR3, but still the signaling preferences of CXCL12 may be altered depending on the presence or the absence of ACKR3; ACKR3 and CXCL12 have higher affinities towards each other compared to their alternative ligands and receptors, respectively [20]. Previously, we established that endothelial cell-derived CXCL12 increases atherosclerotic lesions [14], whereas endothelial CXCR4 is atheroprotective [12]. In our control mice, we established that plasma levels of CXCL12 correlated significantly with lesion sizes, which is also in support of our pro-atherosclerotic findings of endothelial CXCL12. Interestingly, this correlation was lost in the absence of ACKR3 in our EC-ACKR3 deficient mice. There is a possibility that CXCL12 may prefer signaling more heavily via ACKR3 in its presence and when ACKR3 is lost, this signaling may skew more towards the atheroprotective CXCR4. Remarkably, arterial endothelial CXCR4 deficiency leads to increased ICAM + endothelial cells in atherosclerotic lesions as well as increased endothelium–leukocyte adhesion [12], which is the opposite effect in comparison to our arterial endothelial ACKR3 knockout data. The signaling behavior of CXCL12 through CXCR4 and ACKR3 still remains to be studied in greater detail in order to further elucidate these findings. Interestingly, however, SMC-specific roles of CXCR4 and ACKR3 do not seem to be in contrast. These notions indicate that the performance of these receptors may be cell specific.

The functional role of ACKR3 in cell adhesion has been confirmed in different cell lines [11, 28, 37, 56, 68] and it points towards an inflammatory role of this receptor, as also implicated by other studies [41, 64]. ACKR3 was shown to be vital for very late antigen-4 (VLA-4) and lymphocyte function-associated antigen-1 (LFA-1)-driven adhesion of human lymphocytes onto inflamed endothelium [26]. Moreover, ACKR3 knockdown resulted in suppression of adhesion along with VCAM-1 expression in TNF-α-stimulated human brain microvascular endothelial cells [35]. We show for the first time that human ACKR3 in HCAECs regulates the expression of key adhesion molecules ICAM and VCAM under inflammatory conditions. The fact that the role of ACKR3 in cell adhesion is proven across different studies and several cell lines is a strong evidence that this is a general effect of ACKR3. In our study, downregulation of adhesion molecules mediated by ACKR3 silencing occurred concomitant with a decrease in ERK1/2 and Akt phosphorylation as well as a decrease in the phosphorylation of NF-κB p-65 subunit. It has been established that ACKR3 interacts and internalizes with ß-arrestin 2 upon activation, which then acts as a scaffold protein to recruit MAPK proteins to activate ERK1/2 [1, 27, 30, 31, 33, 38, 48]. The impact of ACKR3 on Akt signaling has also been established previously [8, 44, 66]. It is well known that activated Akt can phosphorylate the NF-κB inhibitor IκBα kinase (IIK), leading to the activation of NF-κB [4] and ERK1/2 was shown to activate NF-κB via IκBα, as well [9]. NF-κB is an important transcription factor for ICAM and VCAM and its activation through the degradation of IκBα is well described [34]. Moreover, in an acute pulmonary inflammation murine model, inhibition of ACKR3 was shown to reduce NF-κB phosphorylation [42]. Researchers further confirmed this role of ACKR3 in an acute peritoneal inflammation murine model in which antagonism of ACKR3 diminished the phosphorylation of ERK and NF-κB p-65 [41]. MAPK and NF-κB signaling are well-known inflammatory pathways and ERK/NF-κB signaling was previously shown to induce ICAM and VCAM expression in endothelial cells [67].

Furthermore, we established that ACKR3 silencing increases *PPAR-γ* expression in HCAECs. PPAR-γ is known to be anti-inflammatory and suppressive for NF-κB [45, 50, 54, 63], although such causality is not validated further in our experiments. PPAR-γ activation has also been reported to inhibit ICAM and VCAM expression, which is in line with our results [43, 49]. It is not completely understood how ACKR3 modulates *PPAR-γ* expression; however, we previously established this role of ACKR3 in the adipose tissue of hyperlipidemic mice as well [18]. The fact that ACKR3 deficiency increases *PPAR-γ* expression in different cell types suggests that this is also a general effect of ACKR3. Interestingly, both PPAR-γ and NF-κB have been shown to regulate ACKR3 expression in return, suggesting a circle effect between these molecules. Treatment of human macrophages with pioglitazone to activate PPAR-γ was shown to impede ACKR3 expression resulting in the inhibition of chemotaxis [65]. Others showed that ACKR3 was upregulated in lung squamous cell carcinoma and downregulated by PPAR-γ [51]. In a study investigating the CXCL12/CXCR4/ACKR3 axis in rhabdomyosarcomas, ACKR3 promoter activity was shown to depend on an NF-κB binding site [55]. Collectively, these findings suggest a potential feedback mechanism between these molecules. On the other hand, there is also a possibility that these findings may differ based on the disease model; ACKR3 has been extensively studied in cancer models, whereas our study investigates a cell-specific knockout in the context of atherosclerosis under hyperlipidemic conditions. In conclusion, endothelial-ACKR3 is a novel driver of atherosclerosis and a potential future therapeutic target.

## Supplementary Information

Below is the link to the electronic supplementary material.

Supplementary file1 Supplementary Figure S1: Confirmation of knockout of ACKR3 in mouse models. A. Genotyping of BmxCre mediated deletion of Ackr3 in Apoe-/- mice. The control band is observed at 1.9 kbp and the knockout band is observed at 0.3 kbp. B. Genotyping of SmmhcCre mediated deletion of Ackr3 in Apoe-/- mice. The control band is observed at 1.9 kb and the knockout band is observed at 0.3 kbp. C. Genotyping of Ackr3 deletion in the bone marrow transplantation study. The control band is observed at 1.9 kbp and the knockout band is observed at 0.3 kbp. Supplementary Figure S2: SMC-specific or hematopoietic ACKR3 deficiency does not impact atherosclerosis. A. Schematic representation of the experimental setup (Created with BioRender.com). B. Schematic representation of atherosclerosis prone regions assessed for lesion sizes. C. Representative images (scale bar: 500 µm) and D. quantification of atherosclerotic lesion sizes in the aortic roots (n=8-9). E. Quantification of atherosclerotic lesion sizes in the aortic arches (n=8-9). F. Representative images and G. quantification of atherosclerotic lesion sizes in the abdominal aortas (n=8-9). H. Schematic representation of the 12-week WD experimental setup (Created with BioRender.com). I. Schematic representation of the studied atherosclerosis prone regions. J. Representative images (scale bar: 500 µm) and K. quantification of atherosclerotic lesion sizes in the aortic roots of mice fed with 12 weeks of WD (n=14-16). L. Representative images (scale bar: 500 µm) and M. quantification of atherosclerotic lesion sizes in the aortic arches (n=8-14). N. Quantification of atherosclerotic lesion sizes in the abdominal aortas (n=15-16). Results represent Mean±SEM. Supplementary Figure S3: Further plaque characterization of EC-specific, SMC-specific or hematopoietic ACKR3 deficient mice. A. Quantification of macrophage (MAC2+) content in the aortic roots (n=8-9). B. Representative images (MAC2+ in red and DAPI in blue; scale bar: 250 µm) and C. quantification of macrophages within the aortic root lesions (n=15). D-F. Quantification of SMC (SMA+) content in the aortic root atherosclerotic lesions of SMC-specific (D, n=8-9), hematopoietic (E, n=15), or EC-specific (F, n=12-13) ACKR3 deficient mice. G. Quantification of collagen content in the aortic roots (n=8-9). H. Representative images (scale bar: 250 µm) and I. quantification of collagen content within the aortic root lesions (n=15). Results represent Mean±SEM. *p<0.05. Supplementary Figure S4: Further parameters in endothelial ACKR3 deficient mice. A. Spearman r correlation of lesional SMC content and collagen content in atherosclerotic lesions (r=0.448, p=0.024), (n=25). B. Plasma CXCL12 levels (n=12-14). Results represent Mean±SEM. Supplementary Figure S5: Further results from Ackr3 silenced HCAECs. A. Expression of ACKR3 in control and silenced HCAECs (n=6). B. Confirmation of ACKR3 protein downregulation in silenced HCAECs via western blot. Spearman r correlation of C. ICAM and ACKR3 (r=0.7, p<0.0001) and D. VCAM and ACKR3 (r=0.6, p=0.0039) expression in HCAECS measured by flow cytometry. Western blot images of E. ERK1/2 and F. p-65 NF-kB phosphorylation (phospho protein normalized to total protein) in control and silenced cells. G. Upregulation of ACKR3 expression in ACKR3-transfected HEK cells (n=3). Results represent Mean±SEM. **p<0.01, ***p<0.001 (PPTX 3433 KB)
