# Supplementary material for: Endothelial ACKR3 drives atherosclerosis by promoting immune cell adhesion to vascular endothelium
Source: Basic Res Cardiol. 2022 Jun 8;117(1):30. doi: 10.1007/s00395-022-00937-4 (PMC9177477; doi:10.1007/s00395-022-00937-4)
Supplement: Supplementary file 1 — Supplementary file1 Supplementary Figure S1: Confirmation of knockout of ACKR3 in mouse models. A. Genotyping of BmxCre mediated deletion of Ackr3 in Apoe-/- mice. The control band is observed at 1.9 kbp and the knockout band is observed at 0.3 kbp. B. Genotyping of SmmhcCre mediated deletion of Ackr3 in Apoe-/- mice. The control band is observed at 1.9 kb and the knockout band is observed at 0.3 kbp. C. Genotyping of Ackr3 deletion in the bone marrow transplantation study. The control band is observed at 1.9 kbp and the knockout band is observed at 0.3 kbp. Supplementary Figure S2: SMC-specific or hematopoietic ACKR3 deficiency does not impact atherosclerosis. A. Schematic representation of the experimental setup (Created with BioRender.com). B. Schematic representation of atherosclerosis prone regions assessed for lesion sizes. C. Representative images (scale bar: 500 µm) and D. quantification of atherosclerotic lesion sizes in the aortic roots (n=8-9). E. Quantification of atherosclerotic lesion sizes in the aortic arches (n=8-9). F. Representative images and G. quantification of atherosclerotic lesion sizes in the abdominal aortas (n=8-9). H. Schematic representation of the 12-week WD experimental setup (Created with BioRender.com). I. Schematic representation of the studied atherosclerosis prone regions. J. Representative images (scale bar: 500 µm) and K. quantification of atherosclerotic lesion sizes in the aortic roots of mice fed with 12 weeks of WD (n=14-16). L. Representative images (scale bar: 500 µm) and M. quantification of atherosclerotic lesion sizes in the aortic arches (n=8-14). N. Quantification of atherosclerotic lesion sizes in the abdominal aortas (n=15-16). Results represent Mean±SEM. Supplementary Figure S3: Further plaque characterization of EC-specific, SMC-specific or hematopoietic ACKR3 deficient mice. A. Quantification of macrophage (MAC2+) content in the aortic roots (n=8-9). B. Representative images (MAC2+ in red and DAPI in blue; scale [file 395_2022_937_MOESM1_ESM.pptx]

## Slide 1
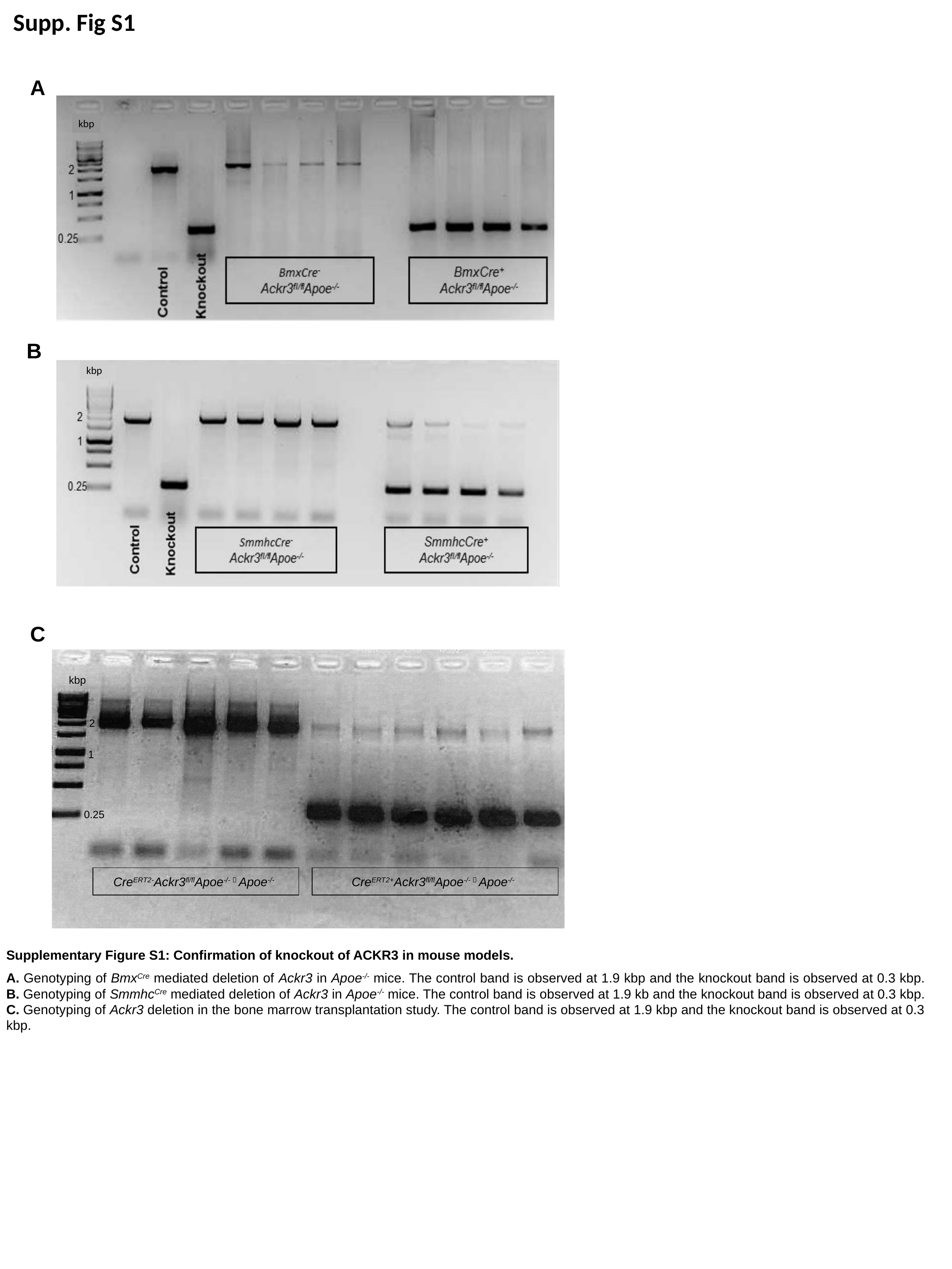

Supp. Fig S1
A
kbp
B
kbp
C
kbp
2
1
0.25
CreERT2-Ackr3fl/flApoe-/-  Apoe-/-
CreERT2+Ackr3fl/flApoe-/-  Apoe-/-
Supplementary Figure S1: Confirmation of knockout of ACKR3 in mouse models.
A. Genotyping of BmxCre mediated deletion of Ackr3 in Apoe-/- mice. The control band is observed at 1.9 kbp and the knockout band is observed at 0.3 kbp. B. Genotyping of SmmhcCre mediated deletion of Ackr3 in Apoe-/- mice. The control band is observed at 1.9 kb and the knockout band is observed at 0.3 kbp. C. Genotyping of Ackr3 deletion in the bone marrow transplantation study. The control band is observed at 1.9 kbp and the knockout band is observed at 0.3 kbp.

## Slide 2
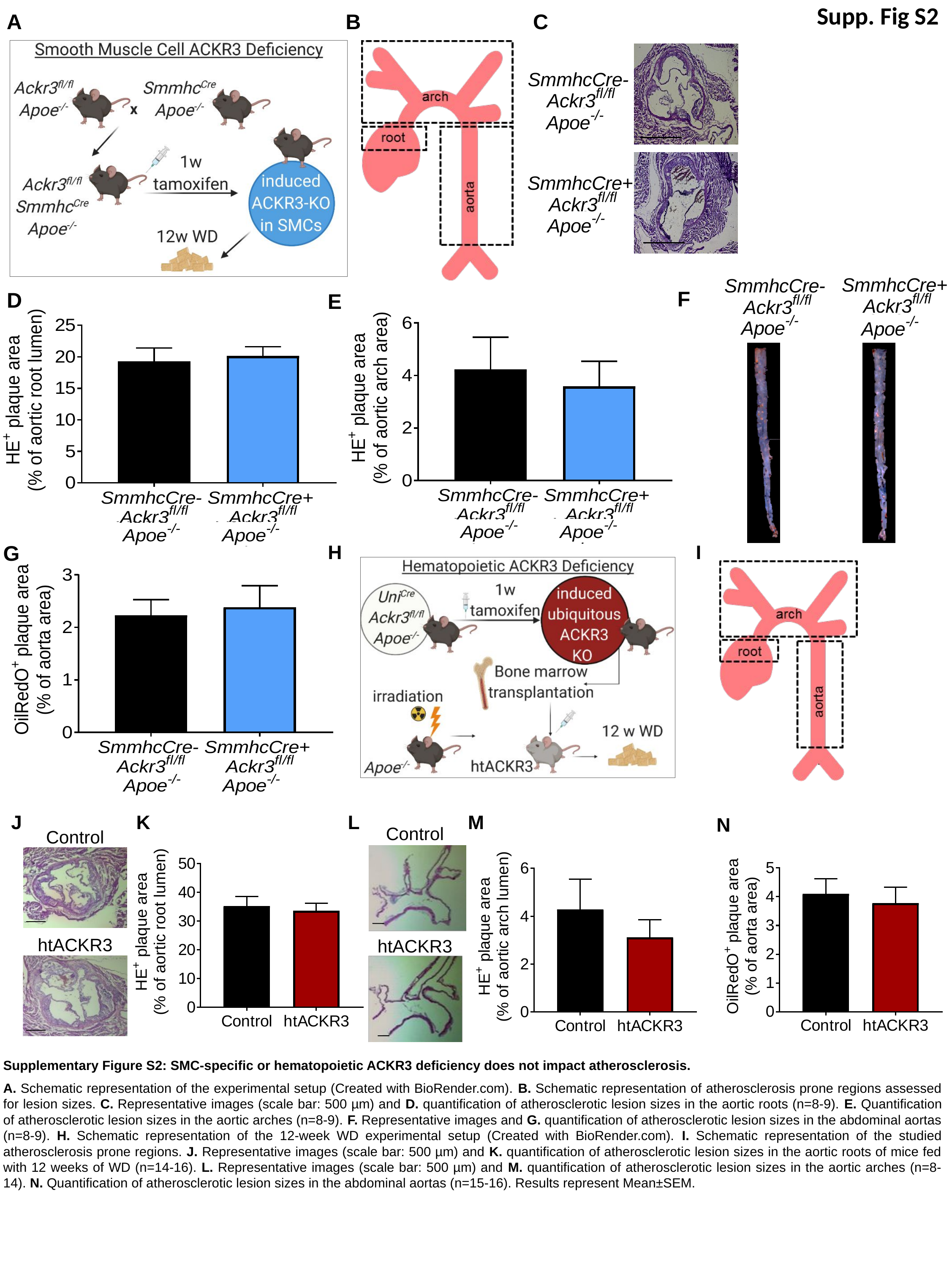

Supp. Fig S2
A
B
C
F
D
E
G
H
I
J
K
L
M
N
Control
htACKR3
Control
htACKR3
Supplementary Figure S2: SMC-specific or hematopoietic ACKR3 deficiency does not impact atherosclerosis.
A. Schematic representation of the experimental setup (Created with BioRender.com). B. Schematic representation of atherosclerosis prone regions assessed for lesion sizes. C. Representative images (scale bar: 500 µm) and D. quantification of atherosclerotic lesion sizes in the aortic roots (n=8-9). E. Quantification of atherosclerotic lesion sizes in the aortic arches (n=8-9). F. Representative images and G. quantification of atherosclerotic lesion sizes in the abdominal aortas (n=8-9). H. Schematic representation of the 12-week WD experimental setup (Created with BioRender.com). I. Schematic representation of the studied atherosclerosis prone regions. J. Representative images (scale bar: 500 µm) and K. quantification of atherosclerotic lesion sizes in the aortic roots of mice fed with 12 weeks of WD (n=14-16). L. Representative images (scale bar: 500 µm) and M. quantification of atherosclerotic lesion sizes in the aortic arches (n=8-14). N. Quantification of atherosclerotic lesion sizes in the abdominal aortas (n=15-16). Results represent Mean±SEM.

## Slide 3
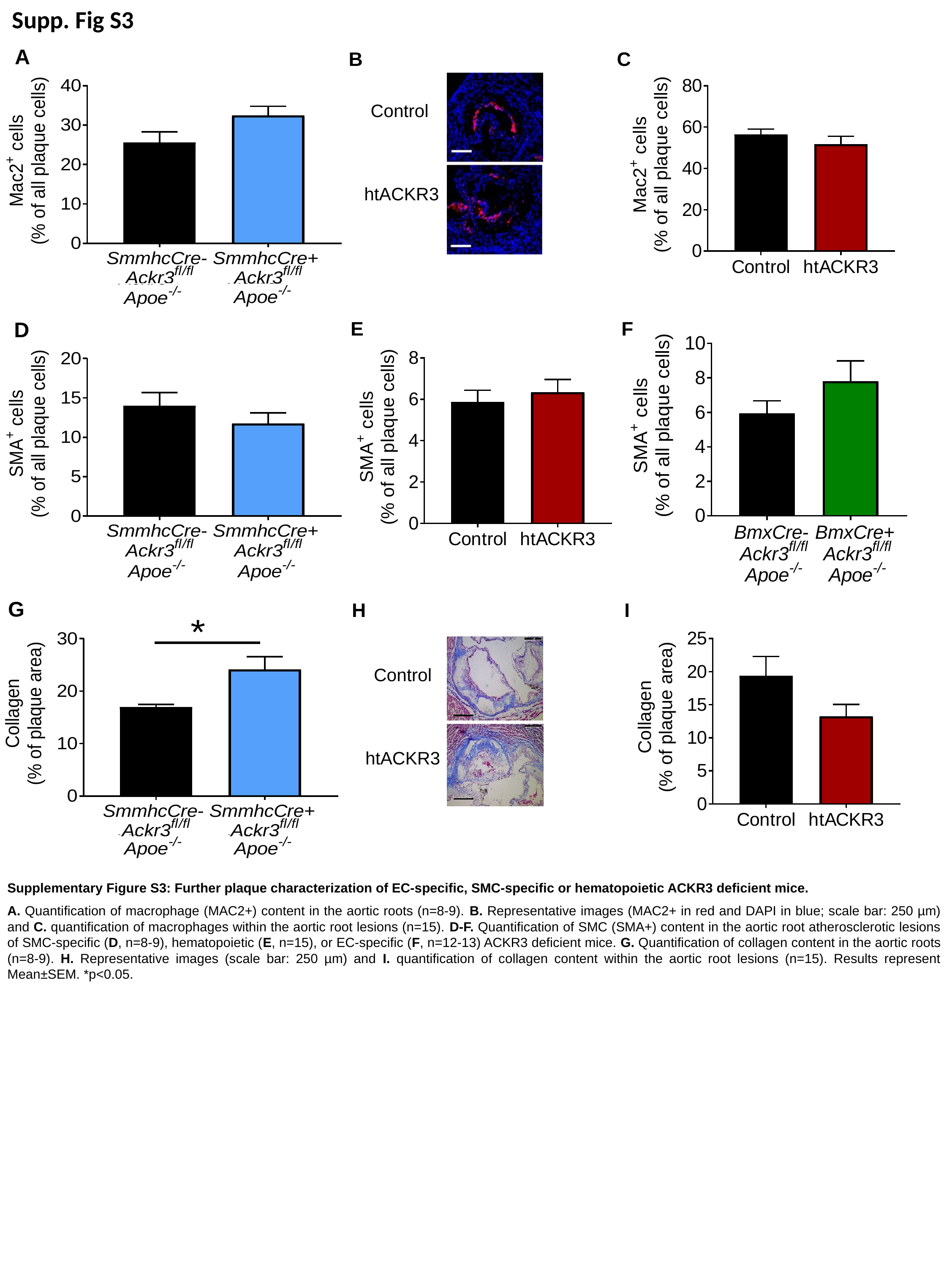

Supp. Fig S3
A
B
C
Control
htACKR3
D
E
F
G
H
I
Control
htACKR3
Supplementary Figure S3: Further plaque characterization of EC-specific, SMC-specific or hematopoietic ACKR3 deficient mice.
A. Quantification of macrophage (MAC2+) content in the aortic roots (n=8-9). B. Representative images (MAC2+ in red and DAPI in blue; scale bar: 250 µm) and C. quantification of macrophages within the aortic root lesions (n=15). D-F. Quantification of SMC (SMA+) content in the aortic root atherosclerotic lesions of SMC-specific (D, n=8-9), hematopoietic (E, n=15), or EC-specific (F, n=12-13) ACKR3 deficient mice. G. Quantification of collagen content in the aortic roots (n=8-9). H. Representative images (scale bar: 250 µm) and I. quantification of collagen content within the aortic root lesions (n=15). Results represent Mean±SEM. *p<0.05.

## Slide 4
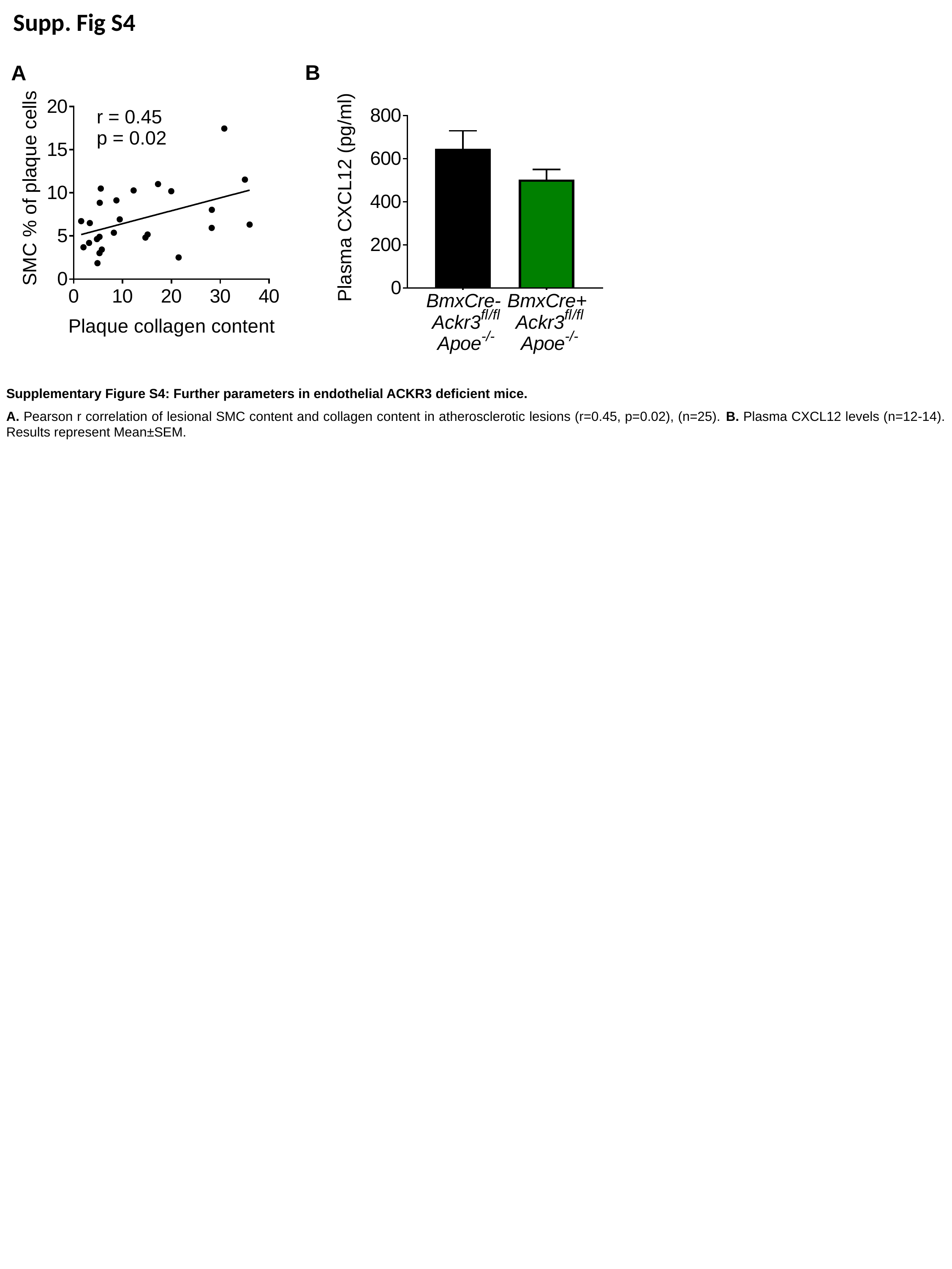

Supp. Fig S4
B
A
Supplementary Figure S4: Further parameters in endothelial ACKR3 deficient mice.
A. Pearson r correlation of lesional SMC content and collagen content in atherosclerotic lesions (r=0.45, p=0.02), (n=25). B. Plasma CXCL12 levels (n=12-14). Results represent Mean±SEM.

## Slide 5
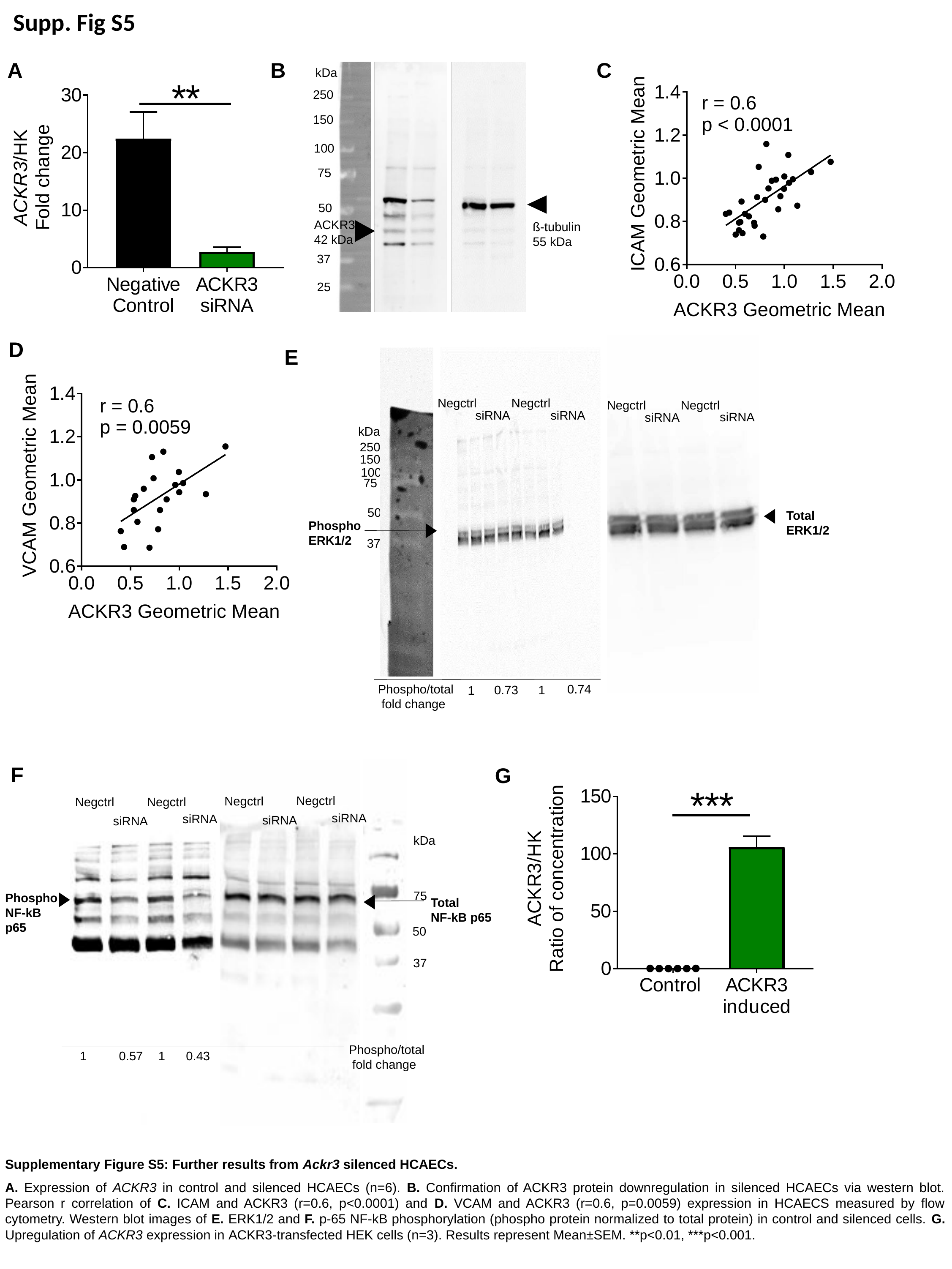

Supp. Fig S5
A
B
C
kDa
250
150
100
75
50
ACKR3
42 kDa
ß-tubulin 55 kDa
37
25
D
Negctrl
Negctrl
Negctrl
Negctrl
siRNA
siRNA
siRNA
siRNA
kDa
250
150
100
75
50
Total
ERK1/2
Phospho ERK1/2
37
0.74
Phospho/total
 fold change
0.73
1
1
E
F
G
kDa
75
50
37
Negctrl
Negctrl
Negctrl
Negctrl
siRNA
siRNA
siRNA
siRNA
Phospho NF-kB p65
Total
NF-kB p65
Phospho/total
 fold change
1
0.57
1
0.43
Supplementary Figure S5: Further results from Ackr3 silenced HCAECs.
A. Expression of ACKR3 in control and silenced HCAECs (n=6). B. Confirmation of ACKR3 protein downregulation in silenced HCAECs via western blot. Pearson r correlation of C. ICAM and ACKR3 (r=0.6, p<0.0001) and D. VCAM and ACKR3 (r=0.6, p=0.0059) expression in HCAECS measured by flow cytometry. Western blot images of E. ERK1/2 and F. p-65 NF-kB phosphorylation (phospho protein normalized to total protein) in control and silenced cells. G. Upregulation of ACKR3 expression in ACKR3-transfected HEK cells (n=3). Results represent Mean±SEM. **p<0.01, ***p<0.001.
